# Balloon Eustachian Tuboplasty: A Systematic Review of Technique, Safety, and Clinical Outcomes in Chronic Obstructive Eustachian Tube Dysfunction

**DOI:** 10.3390/healthcare13151832

**Published:** 2025-07-27

**Authors:** Katarzyna Gołota, Katarzyna Czerwaty, Karolina Dżaman, Dawid Szczepański, Nils Ludwig, Mirosław J. Szczepański

**Affiliations:** 1 Department of Otolaryngology, The Medical Centre of Postgraduate Education, 01-813 Warsaw, Poland; k.golota88@gmail.com (K.G.); katarzynaczerwaty@gmail.com (K.C.); kfrydel@poczta.onet.pl (K.D.); d.szczepanski7@gmail.com (D.S.); 2 Department of Biochemistry, Medical University of Warsaw, 02-091 Warsaw, Poland; nils.ludwig.dmd@gmail.com

**Keywords:** eustachian tube, balloon dilation, tuboplasty, BDET, ETD, ETDQ-7

## Abstract

**Background/Objectives**: Obstructive Eustachian tube dysfunction (OETD) is common in adults and may lead to middle-ear conditions such as atelectasis and cholesteatoma. The ETDQ-7 questionnaire is used to assess symptom severity. Balloon dilation of the Eustachian tube (BDET) is a minimally invasive treatment with variable outcomes. This review evaluates the safety and effectiveness of BDET. **Methods**: A systematic review was conducted following PRISMA 2020 guidelines. Four databases (PubMed, Scopus, Cochrane, Web of Science) were searched using ETD- and BDET-related terms, with the last search on 11 April 2025. Randomized trials were selected based on predefined criteria, and data were extracted by two independent reviewers. Discrepancies were resolved by consensus. **Results**: This systematic review included 14 studies on BDET published between 2013 and 2025. BDET improved otoscopic findings, Valsalva maneuver (VM) performance, and tympanometry (TMM), particularly within the first 6 weeks. ETDQ-7 scores generally indicated symptom improvement, though pure tone audiometry (PTA) showed no significant changes. Most procedures were performed under general anesthesia, with some studies showing similar outcomes under local anesthesia. Combining BDET with other interventions produced mixed results. Reported complications were rare. **Conclusions**: BDET is a safe, low-risk procedure that effectively reduces tympanic membrane retraction and improves VM and TMM results. While it relieves ETD symptoms in many patients, evidence for long-term efficacy and impact on PTA is limited.

## 1. Introduction

Disorders of the Eustachian tube (ET) opening were divided into those resulting from functional obstruction (the most frequent), dynamic disorders, or anatomical obstructions [1]. Obstructive ET dysfunction (OETD) is a common problem in adults, occurring with a prevalence of 0.9% [2]. No universally accepted or effective treatment strategy has been developed [2]. OETD is a relatively frequent otological pathology and, at the same time, a key condition in atelectasis of the middle ear or the formation of acquired cholesteatoma [3]. It is commonly hypothesized that OETD contributes to the development of negative pressure in the middle ear compared to ambient pressure, resulting in atelectasis of the tympanic cavity and the retraction pocket formation. Failure to clear the accumulated keratinized squamous epithelium from the outer layer of the tympanic membrane is believed to be fundamental in the genesis of cholesteatoma [4].

Assessment of ET function should begin with a thorough medical history. Standardized tools like the ETDQ-7 questionnaire aid in symptom evaluation and treatment monitoring. ETDQ-7 consists of seven items addressing common ETD symptoms (e.g., pressure, pain, crackling, muffled hearing), rated on a 1–7 scale. The mean score reflects symptom severity, with ≥14.5 suggesting clinically significant ETD and the need for further evaluation [5].

According to the literature [1], chronic ETD is diagnosed when symptoms persist for more than 3 months. ETD symptoms should be correlated with the test results, beginning with an otoscopic or otomicroscopic examination, followed by objective measurements of negative pressure in the middle ear. Tympanometry is a useful tool in the assessment of Eustachian tube dysfunction; however, the clinical examination remains the most crucial aspect for diagnosing ETD.

There are several available methods for treating ETD, which can be categorized as follows:

1. Conservative management:
  - Autoinflation and maneuvers (e.g., Valsalva, Toynbee): help equalize middle-ear pressure.
  - Nasal therapies (steroids, decongestants, antihistamines): may reduce inflammation, but intranasal steroids show limited benefit (11–18%) in chronic ETD—by Llewellyn et al.
  - Saline irrigation and adjuncts (swallowing exercises, smoking cessation, allergy control): supportive with modest benefits.
2. Medical treatment:
  - Oral steroids or antibiotics: reserved for infection/inflammation; inconsistent evidence for long-term superiority.
3. Procedural/surgical interventions:
  - Myringotomy with tympanostomy tubes provides temporary relief but does not correct ET dysfunction.
  - Emerging techniques (laser/microwave tuboplasty, transtubal tools): promising but require further validation.
  - Balloon Eustachian tuboplasty (BDET/BET).

Balloon dilation of the Eustachian tube (BDET), introduced to clinical practice by Ockermann et al. in 2010 [6], has emerged as a promising treatment for ETD. BDET works by applying pressure to the mucous membrane and soft tissues to promote prolonged ET patency [7]. This minimally invasive endoscopic procedure is the dilation of the cartilaginous portion of the ET through catheterization. Although BDET shows high success rates, a reliable summary of its outcomes is lacking due to varied methodologies among studies [8]. The literature review showed the valuable Cochrane publication [9], partially covering the topic we are investigating, conducted up to 18 January 2024. Our review provides more up-to-date information, searched up to 11 April 2025. Recently, the results of several very interesting, randomized trials have been published, a summary of which seems very welcome. The Cochrane review included nine studies and 684 participants. This manuscript includes 14 studies (6 of which were included in the Cochrane review, 5 of which were not included in the Cochrane review but were published before 2024, and 3 of which were published in 2024 after the Cochrane review’s search date). Cochrane did not evaluate long-term efficacy, subgroup variations, clinical contexts (e.g., anesthesia), or procedural differences. Our review offers recommendations for future research—longer follow-up, standardized protocols, more high-quality RCTs, and methodological consistency.

Thus, this study aimed to conduct an up-to-date systematic review following PRISMA standards to evaluate the technique, safety, and effects of BDET. Therefore, the research question posed in this study is whether BDET offers a clinical advantage over alternative therapies for ETD. This up-to-date review will help clarify the potential of BDET in treating ETD.

## 2. Materials and Methods

### 2.1. Literature Retrieval

**Figure 1:**
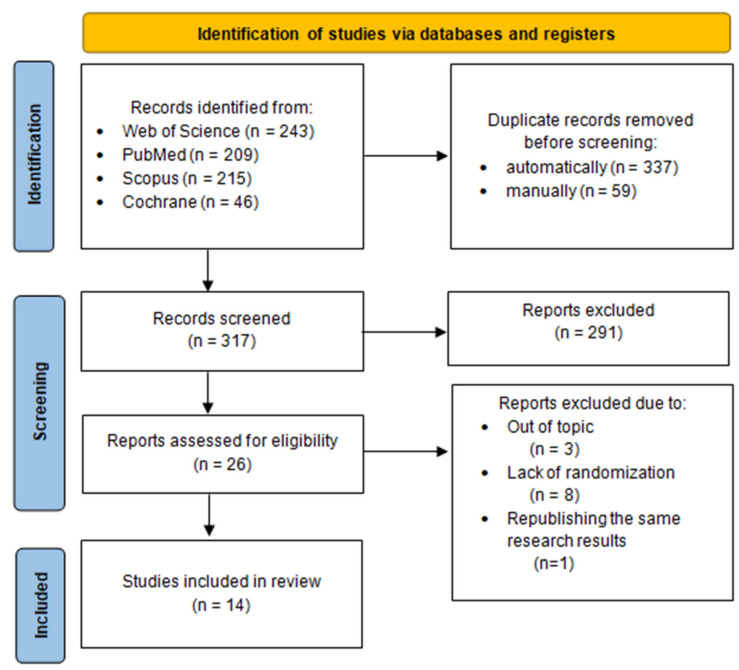
Flow diagram of the systematic literature search. The Preferred Reporting Items for Systematic Reviews and Meta-Analyses (PRISMA) flow diagram shows the study selection process.

This study was conducted according to the Preferred Reporting Items for Systematic Reviews and Meta-Analyses (PRISMA) guidelines, published in 2020 [10]. The literature search process is outlined in the flowchart provided in Figure 1. For this review, we searched the following keywords: “Eustachian tube”, “pharyngotympanic tube”, OR “auditory tube”, AND “dysfunction”, OR “obstruction”, OR “obstructive”, OR “Otitis Media with Effusion”, AND “balloon”, AND “dilation”, OR “tuboplasty”. Four electronic databases were searched (MEDLINE via PubMed, Scopus, Cochrane, and Web of Science) for original articles that included results from randomized trials on balloon Eustachian tube dilation. The search strategy for each database is presented in Table 1.

The last search was performed on 11 April 2025, with no restrictions on the publication date. Additionally, a “snowball” was conducted by reviewing reference lists of eligible full-text articles, but no further studies meeting the inclusion criteria were identified. It was not necessary to contact the authors for additional information. The analysis methods and inclusion criteria were pre-specified, with inclusion and exclusion criteria summarized in Table 2.

Duplicates were removed using the automatic duplicate finder in EndNote 20, followed by a manual check. Eligibility was assessed independently by two reviewers (KCz and KG) in an unblinded, standardized manner. The reviewers screened the titles and abstracts of all retrieved articles, and any disagreements were resolved through discussion to determine which articles required full-text review. Next, three reviewers (KG, KCz, KD) independently assessed the full-text articles for inclusion, again reaching a consensus through discussion if disagreements arose. The results of relevant original studies published in English were summarized and discussed in this systematic review.

### 2.2. Data Extraction

The eligibility of all the studies was evaluated, and the data of each study were retrieved individually by two investigators (KG and KCz), with disagreements resolved using discussion and consensus. These included the following: (1) baseline information, including the first author’s name and year of publication; (2) study design, including BDET effectiveness, technique, and exclusion criteria.

### 2.3. PICO

This study aimed to obtain the following PICO data:

- Population: adult patients with OEDT and/or otitis media
- Intervention: BDET (using a balloon of any size or pressure, conducted under both general and local anesthesia) as a sole procedure or in combination with other treatments (involving nose, paranasal sinuses, and ear)
- Comparison:
  * Comparison with no treatment
  * Comparison with pharmacological treatments
  * Comparison with other methods of surgical treatment
  * Comparison of different techniques of performing the procedurę or the results obtained with the use of local and general anesthesia
- Outcomes (results analyzed at the different time points used in each study—without exclusions):

(1) Primary outcomes:
  * Patient-reported symptom severity and quality of life assessment (using the questionnaires used by the researchers)
  * Results of tonal and impedance audiometry examination
  * Evaluation of adverse events
(2) Secondary outcomes:
  * Otoscopic findings
  * Evaluation of auditory trumpet function using the Valsalva or Toynbee manoeuvre
  * Others were assessed in individual studies, e.g., cost assessment, the graft success rate (in the case of simultaneous myringoplasty), or endoscopic evaluation.

## 3. Results

### 3.1. Search Results

Details of the selection process are summarized in a custom PRISMA flow chart in Figure 1. The systematic literature search retrieved 713 citations from PubMed, Scopus, Cochrane, and Web of Science. Of these, 396 publications were identified as duplicates and were removed. After reviewing titles and abstracts, 291 records were excluded for lack of relevance to BDET or for being review articles, observational studies, or studies without randomization. Full-text verification led to the exclusion of 12 articles (three were off-topic, eight lacked randomizations, and one covered the same results as the article already published and included in our review). Following this screening process, 14 articles met all inclusion criteria and were included in the analysis) [11,12,13,14,15,16,17,18,19,20,21,22,23,24,25].

It should be mentioned that an overlap in patient populations occurred in two of the analyzed studies, specifically those by Poe [23] and Anand [12].

### 3.2. Study Characteristics

Basic data on the research included in this systematic review are summarized in Table S1. All selected articles were original studies published in English. Three studies were conducted in the USA, four in China, two in Korea, and one each in Denmark, Taiwan, the Czech Republic, Finland, and Egypt. The 14 retrieved papers were published between 2013 and 2025.

### 3.3. Risk-of-Bias Assessment

The risk of bias on the main outcomes reported by the included articles was evaluated using the revised Cochrane Risk-of-Bias tool for randomized trials (RoB 2.0). No adaptation to these tools occurred, and no automated tools were used in the evaluation process. The RoB 2.0 tool assessed five predefined areas of bias: (1) randomization process; (2) deviations from intended interventions; (3) missing outcome data; (4) outcome measurement; and (5) selection of the reported outcome. Two independent assessors (DSz and KG) evaluated every study included independently and resolved any discrepancies by discussing and reaching an agreement together.

For the ten RCTs’ intention-to-treat analysis, four studies were considered to be low-risk, three raised some concerns, and three were considered to be high risk of bias mainly because of outcome measurements. Three out of the four studies used in the per-protocol analysis had a low risk of bias, and one study showed some concerns with similar methodological issues.

**Figure 2:**
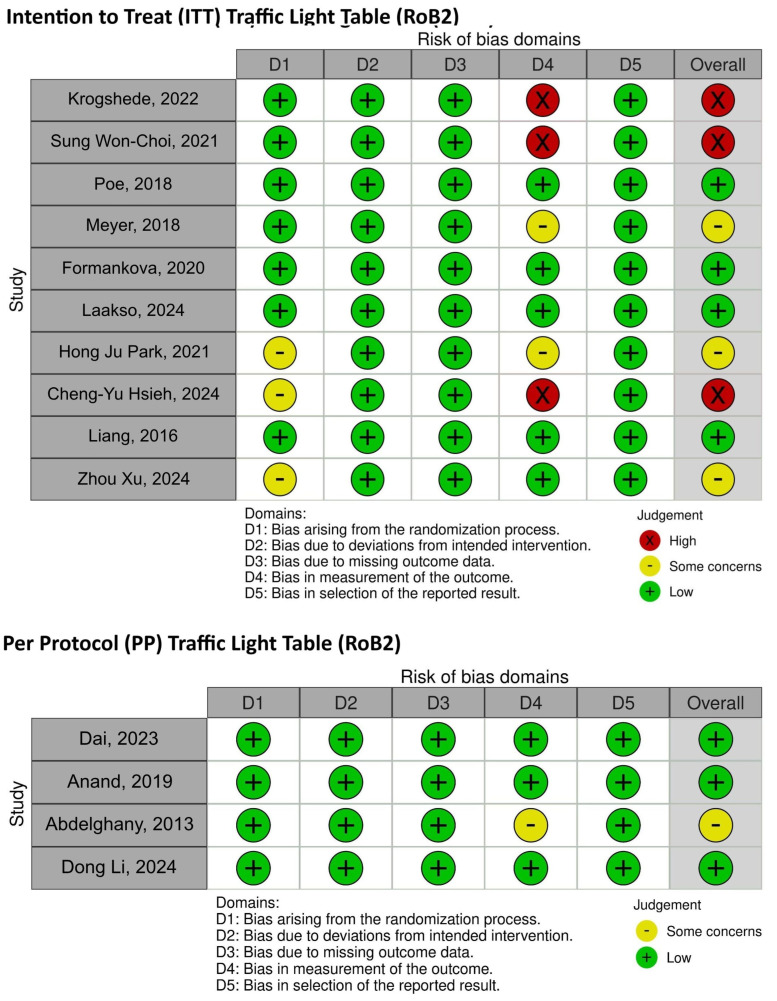
Graphic representations detailing RoB 2.0 [11,12,13,14,15,16,17,18,19,20,21,22,23,25].

**Figure 3:**
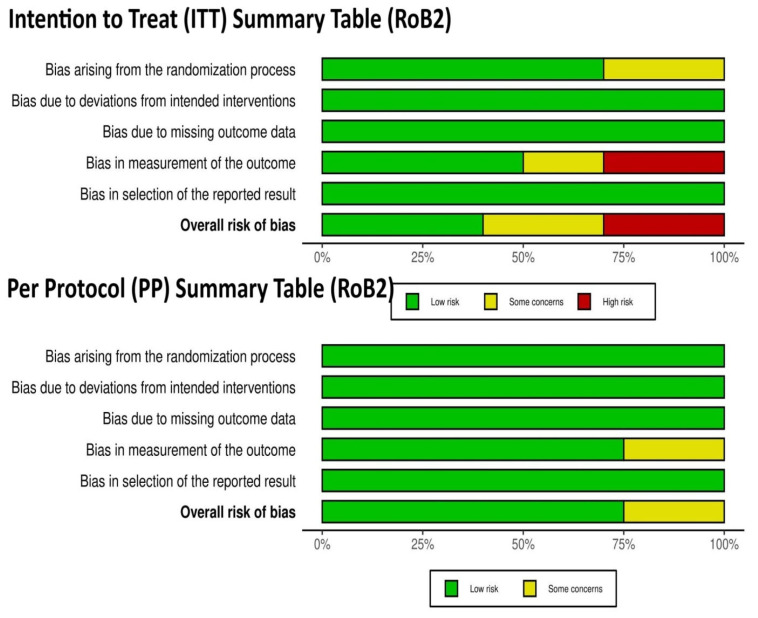
Graphic representations detailing RoB 2.0.

No communication with the authors of the initial studies was required to complete the risk-of-bias assessment exercise. Graphic representations detailing RoB 2.0 are provided in the Supplementary Materials to provide transparency and high methodological quality (Figure 2 and Figure 3).

**Figure 4:**
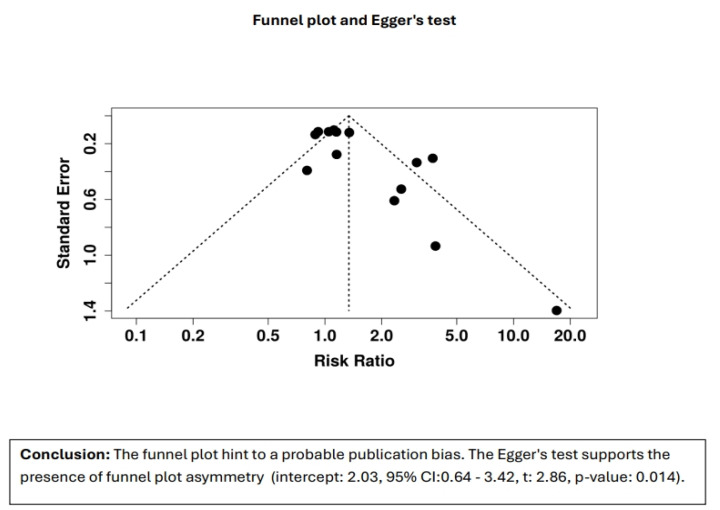
Funnel plot and Egger’s test.

We made a comprehensive evaluation of the likelihood of publication bias with the use of both the funnel plot and Egger’s test. We generated a funnel plot to visually assess the symmetry of the included studies. The plot suggests possible publication bias, as there is noticeable asymmetry, indicating that studies with smaller sample sizes or those showing negative or insignificant effects might be underrepresented in the analysis. We also used Egger’s test to statistically examine the possibility of publication bias. The findings are highly significant in showing asymmetry (intercept = 2.11, 95% CI: 0.75–3.46, t = 3.046, *p*-value = 0.011), supporting evidence to prove the presence of bias among the included papers. The finding is consistent with the funnel plot and shows that the overall findings are probably being influenced by publication bias. Although we carefully followed a structured and comprehensive process for literature inclusion, including systematic searches across multiple databases (PubMed, Scopus, Cochrane, and Web of Science), we acknowledge that the limited number of studies in this specific field may have contributed to the observed publication bias. Despite our rigorous selection criteria, there is still a lack of studies with negative or insignificant results, which could explain the asymmetry in the funnel plot. This might be indicative of a gap in the existing literature rather than a flaw in our study selection process (Figure 4).

### 3.4. Effectiveness of BDET

The effects of BDET were assessed in various ways by different authors. Some relied on otoscopic evaluation of the ears before and after the procedure [14,18,19,21,22] while others compared the results of the Valsalva maneuver (VM) [12,13,15,17,19,21,23] tympanometry [11,12,13,14,15,18,19,21,22,23,24], or pure tone audiometry (PTA) [11,13,15,17,18,19,20], and tubomanometry [19]. Most researchers also examined the impact of BDET on changes in subjective ETD symptoms [12,13,14,15,16,17,18,19,20,22,23,24].

Fibroendoscopic assessment was not incorporated into our systematic review as none of the included studies evaluated BDET outcomes utilizing this method. Consequently, it was not discussed in our manuscript. However, we acknowledge the clinical significance of fibroendoscopy, given its widespread use in numerous clinical centers at present.

#### 3.4.1. Otoscopic and Valsalva Maneuver Results of BDET

In the studies included in the systematic review, all researchers reported improvements in otoscopic results and VM after BDET. In Krogshede’s study [18], the BDET group showed normalization from retraction or serous otitis media in 9 out of 13 patients (69%). Similarly, Poe [23] observed a 32.8% increase in the number of ears with a positive modified VM at a 6-week follow-up. Hong Ju Park [17] combined BDET with medical management (MM), achieving an even higher success rate in the VM (46.8% of ears). In this study, patients received 8 weeks of medical management (MM) before BDET. They were evaluated via Valsalva, PTA, SA, and ETDQ-7, and those with persistent dysfunction could cross over to BDET. Post-procedure, they followed the same follow-up protocol as the initial BDET group, enabling assessment of BDET’s added benefit over MM alone.

Choi [13] reported a more modest yet statistically significant improvement (*p* = 0.014), with 31.6% (6/19) of cases showing a positive modified VM in the BDET group compared to 15.8% (3/19) in the control group at a 6-week follow-up.

Several studies compared short- and long-term outcomes of BDET. Anand [12] observed similar results at 6- and 52-week follow-ups, with 78.6% of ears showing a positive VM after 6 weeks and 80.4% after 52 weeks. Consistent with Anand’s findings, other researchers [15,23] also reported BDET results at both 6-week and 12-month follow-ups. Among participants with retracted TMs at baseline, 66.7% showed improved TM position after 6 weeks, and 47.1% converted from a negative to a positive VM. At the 12-month follow-up, over 80% of participants demonstrated a normal TM position, and the number of ears with a positive VM had doubled.

However, a multicenter, double-blind, randomized study conducted over 12 months on 20 ears found comparable reductions in otoscopy and VM results between active and sham groups [19].

In summary, most studies indicate that BDET reduces TM retraction and improves VM performance, typically observed within 6 weeks of treatment. These improvements generally increase over longer follow-up periods, though the degree of improvement varies across studies, ranging from 30% to 80%.

#### 3.4.2. The Pure Tone Audiometry and Tympanometry Results of BDET

None of the analyzed studies reported a statistically significant change in PTA following BDET when used as a standalone procedure [13,17,18,19,22]. Shifts in PTA from baseline to the 6-month follow-up were not statistically significant at any measured frequency.

All investigators [12,13,18,22,23] confirmed short-term benefits (6 weeks post-procedure) from BDET, evidenced by improved tympanometry results in 36–69% of cases, with most studies reporting improvements in 50–60% of participants. For example, Krogshede [18] found tympanometry improvement in 69% (9/13) of patients in the BDET group (showing shifts from B to C/A or from C to A) compared to 27% (3/11) in the control group (*p* = 0.04). However, some studies reported more modest results. Choi [13] observed tympanogram changes from baseline in 36.8% of ears in the BDET group compared to 15.8% in the CG, with improvements shifting from B or C2 to C1 or A or from B to C2. Several researchers compared short- and long-term outcomes [12,22,23], noting tympanogram normalization at a 6-week follow-up in approximately 51–57% of cases, increasing to 62% [12,23] or even up to 80% after a 52-week follow-up [22].

In summary, BDET provided significant improvements in tympanometry assessments for participants with abnormal middle-ear function at baseline, with these benefits increasing over longer follow-up periods. However, BDET did not achieve improvements in PTA outcomes.

#### 3.4.3. The ETDQ-7 Results of BDET

The ETDQ-7 score is particularly valuable for assessing treatment effectiveness (e.g., medical therapy, BDET) or tracking symptom changes over time in patients with chronic or recurrent ETD. Several studies have evaluated ETDQ-7 score changes following BDET for ETD. Most reported a significant (nearly twofold) reduction in ETDQ-7 scores post-operatively [13,17] with BDET patients showing significantly greater symptom improvement at 6 weeks compared to the CG [13,22,23]. However, two studies [18,19] found no significant difference in mean ETDQ-7 scores between the BDET and CGs.

Regarding long-term BDET outcomes, Anand [12] reported ETDQ-7 scores below 2.1 in 55.6% of participants after 6 weeks and 57.3% after 52 weeks. Other researchers [22] noted a significant reduction in the mean ETDQ-7 score from 4.6 at baseline to 2.1 at 6 weeks, with this reduction maintained through a 12-month follow-up.

These findings suggest that BDET generally improves ETD symptoms compared to CG, though not all studies confirmed this effect.

### 3.5. Safety and the Main Exclusion Criteria for the BDET Technique

BDET is a minimally invasive procedure used to treat Eustachian tube dysfunction. Studies indicate that BDET is generally safe, with complication rates of about 2%. In none of the included studies were significant complications following BDET reported. The authors underlined that navigation-guided BDET is a safe procedure [12]. However, Hussain [26] described potential complications following BDET, categorizing them into major and minor complications. Major complications include emphysema and pneumomediastinum. Minor complications observed were bleeding, hemotympanum, transient tinnitus, OMS, rhinitis, altered tongue sensation due to chorda tympani compression, dizziness, and acute otitis media. McCoul [5] reported a 2% complication rate associated with BDET, with surgical emphysema well documented in the literature, occurring in approximately 0.27% of cases [5,27]. The study highlights that the success of BDET depends heavily on careful patient selection. Trauma to the ET mucosa during catheter positioning can lead to post-operative emphysema. Additionally, catheter material plays a significant role; rigid catheters can be challenging to insert, risking mucosal damage and increasing the chance of complications. Cracks within the ET are noted to possibly result from the use of inelastic catheters [26]. Successful outcomes depend on careful patient selection and the use of flexible catheters to minimize mucosal injury and complications [26].

The catheter tip is considered a potential source of infection [28] as it often carries a variety of bacteria that normally colonize the throat mucosa, including *Corynebacterium*, *Staphylococcus aureus*, *Staphylococcus epidermidis*, *Streptococcus pyogenes*, and *Klebsiella oxytoca*. Antibiotic prophylaxis is recommended if there is any damage to the ET mucosa, as such injury can allow these bacteria to spread to surrounding soft tissues, increasing the risk of infection [27,28].

For post-operative care, most patients are advised to perform the VM immediately following the BDET procedure [27]. However, it was noted that performing the maneuver immediately after BDET might often lead to emphysema [27]. Hussain [26] reported that patients who were instructed to perform VM after the procedure developed extensive subcutaneous emphysema immediately after its performance. Therefore, the author recommended a 2-week period of refraining from performing the VM. Some authors recommended an even longer delay of 3 weeks before performing VM [27]. Additionally, patients should also be informed about contraindications such as weightlifting and excessive physical exertion during the recovery period [26].

Carotid canal dehiscence confirmed in a CT scan was listed as the most important exclusion criterion in all papers. Other exclusion criteria for BDET typically included are as follows:

1. Active infection: acute otitis media or any active infection in the middle ear or sinus (all papers).
2. Allergic rhinitis or uncontrolled sinusitis [22].
3. Severe nasopharyngeal pathology: structural abnormalities or tumors in the nasopharynx (most papers).
4. Cleft lip and palate (most paper).
5. Cystic fibrosis or primary ciliary dyskinesia [12,18,23].
6. Patulous ET: a condition where ET stays open too much, which BDET cannot treat effectively (all papers).
7. Subtotal TM perforation [12,18,19,20,22,23].
8. Cholesteatoma [13,18,19,23].
9. Meniere’s disease [12,23].
10. Preoperative air-bone gap (ABG) above 30 dB 20.
11. Fungal otitis externa [20].
12. Temporomandibular joint disorder [12,23].
13. Gastroesophageal reflux disease [19,22].
14. Pregnancy [13,15,16,17].
15. Major head or neck surgery (most papers).
16. Prior head and neck radiation: radiotherapy affecting healing and procedure outcomes (most papers).
17. Age criteria [12,13,16,19,23].
18. Smoking [19].

### 3.6. Kind of Anesthesia During BDET Procedure

In most studies, the BDET procedure was performed transnasally under general anesthesia (GA) [11,13,15,16,19,20,21,23]. In one instance, only local anesthesia (LA) was used [17], achieved by placing surgical pledges soaked in normal saline with 4% lidocaine and 1:1000 epinephrine into the nasal cavity and over the nasopharyngeal opening of the ET for 5 min under a 0° endoscope view [17]. In the remaining three studies, either LA or GA was offered as an option, while in two others [12,18], no information on the type of anesthesia was provided.

Procedures conducted in the office under LA were well tolerated; however, further research should aim to minimize pain and discomfort. Additionally, they observed that operative time and treatment costs were lower in the LA group compared to the GA group.

### 3.7. BDET Technique

The BDET technique described in all papers was similar. An insertion instrument was positioned near the ET opening, and a balloon catheter was advanced 2 cm into the ET, and then inflated with sterile water to a pressure of 10–12 bar for 2 min. The inflation rate was constant at approximately 1 atm/s to prevent rapid changes in middle-ear pressure, which could cause otalgia and vertigo. Balloons were available in diameters from 3.28 to 7 mm and lengths of 8 and 20 mm. Following dilation, the balloon was deflated, and the catheter was removed. After the procedure, the pharyngeal ostium of the ET appeared smooth, and the openings were enlarged without any tearing. Some authors also used an image-guided navigation system [13].

### 3.8. BDET as Additional Procedure

Some investigators [14,15,21] have examined the benefit of adding BDET as an adjunct procedure alongside tympanotomy tube insertion or paracentesis for treating refractory otitis media secretory (OMS). They [14] observed no differences in TM movements, ETDQ-7 results, and post-operative VAS scores between the groups with and without additional BDET.

Liang [21] compared treatment protocols across three groups: BDET only (group I), BDET with paracentesis group (group II), and paracentesis-only (group III). Both the BDET-only and BDET-plus-paracentesis groups achieved better outcomes than the paracentesis-only group. At one month post-operatively, the BDET-plus-paracentesis group showed improved otic endoscopic scores compared to the BDET-only group (*p* < 0.05), though no significant differences were seen at months 3 or 6. Tympanograms showed no significant intergroup differences at any post-operative check-up. It is important to mention that BDET groups achieved better outcomes compared to the paracentesis-only group. Similar results were obtained by other researchers [15]. They concluded that there was no beneficial outcome of tympanocentesis performed concurrently with BDET and, therefore, it should not be routinely recommended.

Abdelghany [11] assessed the impact of additional BDET in patients undergoing myringoplasty and found significantly better middle-ear pressure at 6 and 12 months post-operatively compared to myringoplasty alone. Improvement in the ETDQ-7 score was observed at 3 months post-operatively, although there was no significant difference between groups at 12 months in ETDQ-7 scores or graft success rate [20].

Another study [16] combined BDET with ESS (endoscopic sinus surgery) for patients with chronic rhinosinusitis (CRS) complicated by ETD, reporting that additional BDET significantly improved ETDQ-7 scores, tympanometry results, and the frequency of a positive VM compared to ESS alone.

In summary, BDET appears beneficial for treating OMS and is more effective than paracentesis alone. It also shows benefits in CRS patients undergoing ESS. However, combining BDET with myringoplasty does not seem to provide long-term advantages.

## 4. Discussion

BDET was introduced into clinical practice as a novel treatment approach for ETD. The concept of BDET involves applying pressure to the mucosa and soft tissues to achieve prolonged ET patency [7]. Our systematic review revealed that BDET is a safe technique associated with a high success rate; however, the qualification criteria for the procedure have not yet been clearly defined. Scientists also differ in the selection of methods for assessing the effects of treatment with BDET, the selection of patients, and the methods for assessing results, which makes it difficult to compare the results achieved by different studies. Several researchers have reported that the ETDQ-7 is a valid and reliable symptom-based questionnaire for use in adult patients with Eustachian tube dysfunction (ETD). It may serve as a useful tool in clinical practice by emphasizing the impact of ETD on patient-reported outcomes. However, additional studies are required to fully establish its utility in evaluating treatment efficacy. Although the ETDQ-7 has been validated for the diagnosis of ETD, its effectiveness in monitoring response to treatment remains insufficiently validated. In the current patient cohort, the ETDQ-7 successfully identified the presence of ETD, but did not reliably detect clinical improvement following intervention.

The study by Poe [23], which included the largest group of patients and simultaneously compared the effectiveness of pharmacological treatment (MM) with the BDET procedure—without using other surgical methods—seems to be the most objective in terms of evaluating the effectiveness of BDET. The results indicated that tympanogram normalization at the 6-week follow-up was achieved in 51.8% (72 out of 139) of patients in the treatment group, compared to 13.9% (10 out of 72) in the control group (*p* < 0.0001). By week 24, the normalization rate in the treatment group increased to 62.2%. Moreover, normalization of ETDQ-7 symptom scores at the 6-week follow-up was observed in 56.2% (77 out of 137) of treated patients, in contrast to 8.5% (6 out of 71) in the control group (*p* < 0.001). In addition, patients in the investigational group showed marked improvement in both mucosal inflammation and Valsalva maneuver (VM) performance at the 6-week follow-up, when compared to controls.

The durability of results following a 2 min dilation of the cartilaginous ET is promising. Since the ET lumen is flexible and not uniformly rigid—except near the isthmus, the exact mechanism of action remains unclear. It appears that the non-compressible balloon may provide similar benefits to adenoidectomy within the ET lumen, where traditional techniques cannot address adenoid-like tissue effectively.

Balloon Eustachian tuboplasty (BDET) is traditionally performed under general or local anesthesia using a dedicated, single-use, non-compressible balloon catheter, with an average procedural cost of approximately $6000 USD per patient. To minimize material and device-related expenses, the use of endovascular balloons (EVBs) for BDET—an off-label application—has been systematically investigated for both feasibility and safety, beginning with cadaver studies and subsequently extending to human subjects. Performing BDET with EVBs under local anesthesia offers the potential for further cost reduction without compromising procedural integrity. We must emphasize that the use of unapproved endovascular balloons (EVBs) must be explicitly characterized as experimental and off-label [29].

Multiple specialized balloon dilation systems used for BDET are classified as Class II medical devices and have received FDA approval. These single-use instruments typically cost over $1000 USD each. In contrast, the mass production and widespread availability of endovascular balloons (EVBs) have reduced their unit cost to approximately $170 USD. When combined with the reduced need for operating room resources, particularly when BDET is performed under local anesthesia in an outpatient setting, this cost differential can result in substantial overall savings, independent of the healthcare delivery model. This economic advantage becomes particularly relevant when repeat procedures are considered for recurrent or persistent symptoms [30].

The systematic review has several limitations. Firstly, most of the included studies had small sample sizes and were single-center studies [12,13,14,17,18,21,31]. The review was limited to studies published between 2013 and 11 April 2025; all selected articles were original studies published in English. Furthermore, many studies did not exclusively assess patients treated with BDET alone. In several cases, BDET was combined with another therapeutic intervention [10,12,13,14,15,19,20,31] most commonly involving an additional procedure on the TM [10,13,14,19].

Additionally, it can be observed that in two of the analyzed studies, the patient groups overlapped—specifically in the studies by Poe [23] and Anand [12]—which constitutes another limitation of the presented review.

Ultimately, the short follow-up period in some study groups—such as 8 weeks [12] or 12 weeks [15] after the procedure—further limits the reliability of the results. Another limitation of the publications is that the maximum follow-up period for patients was one year. Therefore, the data presented in the article reflect outcomes only up to 52 weeks after the BDET procedure.

Our review highlights a gap in the literature, as it would indeed be desirable to conduct studies showing the long-term effects of balloon eustachian tuboplasty.

In this systematic review, we have focused on studies conducted on adults, since in adulthood the anatomy of the nasopharynx, paranasal sinuses, and ET is already formed, and anatomical conditions do not change as dynamically as in children. Nevertheless, several studies—including the systematic review and meta-analysis by Aboueisha et al.—have investigated BDET in children:

- Included seven studies involving 408 children, mean age 9.9 years, with average follow-up of ~19 months. Type B tympanograms decreased from 64.2% to 16.1%.
- Air-bone gap (ABG) improved from 25.3 dB to 10.2 dB.
- Adverse events occurred in ~5.1%, mostly self-limited epistaxis.
- In three studies comparing ventilation tubes, BDET showed superior ABG reduction (mean difference −6.4 dB, *p* = 0.002)

These pediatric data demonstrate that BDET is effective in improving middle-ear function and symptoms, with a favorable safety profile.

Moreover, due to the small groups, it was desired to exclude statistically significant differences in smoking status and GERD, as these are known factors that may promote the persistence of mucosal inflammation in the nasopharynx and possibly affect treatment outcomes. Although there is a lack of reports in the literature on the effect of smoking on Balloon Eustachian Tuboplasty outcomes and it would seem interesting to conduct comparative studies of treatment outcomes between smoking and non-smoking patients.

## 5. Conclusions

In summary, BDET aims to prevent, reverse, or halt disease progression by dilating the cartilaginous portion of the ET thereby improving its function. BDET is a procedure that yields satisfactory outcomes, encouraging its use in a broader population of patients suffering from ETD.

This systematic review provides evidence of the beneficial effects of BDET. Most studies indicate that BDET reduces TM retraction and enhances VM performance. Significant improvements were observed in TMM results following BDET. Overall, BDET leads to better outcomes in ETD symptoms when compared to CG. Additionally, the BDET procedure appears to be safe for the patient, associated with a low risk of complications.

Nevertheless, we must emphasize that the studies included in our systematic review report outcomes only up to 52 weeks following the procedure. Therefore, it is difficult for us to predict the results beyond one year after BDET, as there is currently insufficient evidence to support such long-term conclusions.

## Figures and Tables

**Table 1:** Search strategy for used databases.

Database	Number of Results	Search Strategy
Pubmed	209	((„Eustachian tube” OR „pharyngotympanic tube” OR „auditory tube”) AND („dysfunction” OR „obstruction” OR „obstructive”)) OR “Otitis Media with Effusion” [Mesh]) AND („balloon” AND („dilation” OR „tuboplasty”))
Scopus	215	TITLE-ABS-KEY ((((((eustachian AND tube) OR (pharyngotympanic AND tube) OR (auditory AND tube)) AND ((dysfunction) OR (obstruction) OR (obstructive))) OR (otitis)) AND ((balloon) AND ((dilation) OR (tuboplasty))))) AND (LIMIT-TO (LANGUAGE, “English”))
Cochrane	46	#1 eustachian tube OR pharyngotympanic tube OR auditory tube #2 dysfunction OR obstruction OR obstructive #3 otitis #4 balloon #5 dilation OR tuboplasty #6 ((#1 AND #2) OR #3) AND (#4 AND #5) And Trials
Web of Science	243	TS =(((((eustachian AND tube) OR (pharyngotympanic AND tube) OR (auditory AND tube)) AND ((dysfunction) OR (obstruction) OR (obstructive))) OR (otitis)) AND ((balloon) AND ((dilation) OR (tuboplasty))))

**Table 2:** Inclusion and exclusion criteria.

Inclusion Criteria	Exclusion Criteria
Original articles presenting the results of balloon dilation of the eustachian tube (including case series in people who have previously been treated medically or surgically)	Conference papers and abstracts, reviews, editorials, case reports, retrospective studies, opinions, or letters
Randomized controlled trials (by patient and by ear)	Studies involving patients with nasopharyngeal malignancy and radiation therapy of the head and neck
Human study	Animal study
Study on adults	Study on children
Full-text articles available in English	Language other than English, only abstracts available

## Data Availability

Available on request from the corresponding authors.

## Supplementary Materials

The following supporting information can be downloaded at https://www.mdpi.com/article/10.3390/healthcare13151832/s1, Table S1: Basic characteristics of the studies included in the systematic review.

## Abbreviations

ABG	air-bone gap
BDET	balloon dilation of the Eustachian tube
CG	control group
CNM	cartilage underlay myringoplasty
COME	chronic otitis media with effusion
CRS	chronic rhinosinusitis
ESS	endoscopic sinus surgery
ET	Eustachian tube
ETS	Eustachian tube score
ETD	Eustachian tube dysfunction
ETDQ-7	Eustachian Tube Dysfunction Questionnaire-7
EVB	endovascular balloon
GA	general anesthesia
LA	local anesthesia
MM	medical management
OETD	obstructive Eustachian tube dysfunction
OMS	otitis media secretory
PRISMA	Preferred Reporting Items for Systematic Reviews and Meta-Analyses
PTA	pure tone audiometry
RCT	randomized controlled trial
SNOT-22	sinonasal outcome test 22
TBI	tympanotomy tube insertion
TM	tympanic membrane
TMM	tympanometry
VAS	visual analog scale
VM	Valsalva maneuver
