# Supplementary material for: Balloon Eustachian Tuboplasty: A Systematic Review of Technique, Safety, and Clinical Outcomes in Chronic Obstructive Eustachian Tube Dysfunction
Source: Healthcare (Basel). 2025 Jul 27;13(15):1832. doi: 10.3390/healthcare13151832 (PMC12345958; doi:10.3390/healthcare13151832)
Supplement: Supplementary file 1 [file healthcare-13-01832-s001.zip › healthcare-3674332-supplementary.pdf]

ARTICLE	Type of study	Study Demographics	Methods evaluation	Results	Conclusion
Krogshede[18]	prospective RCT	n= 26 patients:<br>• BDET (n=13)<br>• CG (n=13)<br>Follow-up:<br>3 w., 12 w., 24 w.	1) otomicroscopy<br>2) TMM<br>3) PTA<br>4) ETDQ-7	1) otomicroscopy normalization<br>BDET(9/13) vs. CG (0/11) (p =0.0006)<br>2) TMM improvement:<br>BDET(9/13) vs. CG (3/11) (p =0.04)<br>3) the audiometric data:<br>no improvement (p=0.38)<br>4) ETDQ-7 results<br>BDET vs. CG (p =0.35)	1) BDET is feasible.<br>2) No complications were reported.<br>3) BDET may be beneficial in a selected group of adult patients with mild chronic ETD.
Dai[14]	prospective RCT	n=40 patients (RSOM treated with BDET+TBI):<br>• LA/sedation group (n=20)<br>• GA (n=20)<br>Follow-up:<br>1 w.,3 w., 6 w., 48w.	1) TMM,<br>2) ETDQ-7<br>3) VAS<br>3) side effects<br>4) costs	1) LA group exhibited intraoperative awareness and pain but lower postoperative discomfort (p=0.049).<br>2) TMM, ETDQ-7, VAS scores:<br>no group differences (p>0.05).<br>3) operative time LA vs. GA:<br>49 min. vs 125 min. (p<0.0001).<br>4) costs: lower in LA vs. GA (p<0.0001).	The treatment effects and safety of LA and GA groups were comparable.
Sung-Won Choi[13]	prospective RCT, multicenter, 1:1 parallel-group	n=38 ears:<br>•BDET (n=19 ears)<br>•CG (n=19 ears)<br>Follow-up:<br>6 w.	1) ETDQ-7<br>2) TMM/VM<br>3) PTA	1) ETDQ-7, BDET vs. CG<br>1.99 vs. 3.40 (p= 0.001).<br>2) TMM/VM improvement,<br>BDET vs. CG<br>36.5% vs. 15.8% (p= 0.014)<br>3) ABG decrease:<br>higher in BDET vs. CG (p=0.037)	Navigation-guided BDET is a safe and superior option compared to MM alone in patients with chronic ETD.

Poe[23]	prospective RCT, multicenter	n= 462 ears:<br>• lead-in (n=115 ears),<br>• BDET (n= 234 ears),<br>• MM (n=117 ears)<br><br>Follow- up:<br>6 w., 24 w.	1) ETDQ-7<br>2) TMM/VM	1) TMM normalization BDET vs. CG at 6 w.: 51.8% vs. 13.9% (p<0.0001)<br>at 24 w. 62.2% vs. 13.9%<br>2) normalization of ETDQ -7 at 6w. BDET vs. CG<br>56.2% vs. 8.5%(p<0.001)<br>3) Comparable improvement in both mucosal inflammation and VM at 6 w.	This study demonstrated the superiority of BDET+ MM compared to MM alone
Anand[12]	prospective cohort study, the treatment group - reported in multicenter RCT	n=465 ears:<br>• BDET+MM(n=235)<br>• MM only (n=115)<br>• nonrandomized lead (n=115)<br>Follow-up:<br>6 w., 52 w.	1) ETDQ-7<br>2) TMM/VM	1) TMM normalization and ETDQ7: no difference between groups<br>2) improvement in 6w.vs 52w:<br>TMM: 51.0%vs. 55.5%;<br>ETDQ-7: 55.6% vs. 57.3%<br>VM: 78.6% vs. 80,4%	The beneficial effects of BDET+MM on TMM and ETDQ-7 demonstrate durability through 52 w.
Meyer[22]	Prospective RCT, multicenter,<br><br>5 US centers	n=60 patients:<br>• BDET (n= 31)<br>• MM (CG n=29)<br>Follow-up:<br>6 w., 52 w.<br>Anesthesia:<br>LA (72%)<br>GA (28%)	1) ETDQ-7,<br>2) TMM/VM<br>3) otoscopy	1) ETDQ-7 improvement in 6 w.<br>BDET vs. CG<br>2.9 vs. 0.6 (p < 0.0001).<br>ETDQ improvement maintenance in 52w.<br>2) TMM improvement<br>BDET > CG (p < 0.006)<br>3)VM improvement<br>BDET > CG (p < 0.001)	BDET is a safe and effective treatment for persistent ETD, superior to continued MM in ETDQ-7 improvement. Procedures are well tolerated in the office setting under LA.
Liang[21]	Prospective RCT	n=90 patients<br>• BDET only (n=30)<br>• BDET+paracentesis (n=30)<br>• paracentesis only (n=30)<br>Follow-up:<br>6 w., 12 w., 24 w.	1) ETDQ-7,<br>2) TMM/VM<br>3) otoendoscopy	1) otoendoscopy improvement.<br>BDET+paracentesis>BDET only significant difference only in short time follow-up (6 w.)<br>2) TMM improvement<br>observed only in BDET+paracentesis and BDET only groups	The combination of BDET and paracentesis was effective for intractable COME and made recovery period shorten.

Formankova [15]	RCT	n= 30 ears<br>• BDET only (n=15)<br>• BDET+paracentesis (n=15)<br>Follow-up:<br>2w.,6 w., 48 w.	1) TMM/VM or Toynbee maneuver<br>2) ETDQ - 7<br>3) PTA	No significant difference in the effect of treatment was found between the groups.	BDET should not be routinely recommended.
Laakso[19]	RCT, multicenter, double-blinded	n= 20 ears:<br>• BDET (n=14)<br>• CG (n=6)<br>Surgery under LA<br>Follow-up:<br>3w., 48 w.	1) ETDQ – 7<br>2) TMM/VM or Toynbee maneuver<br>3) otomicroscopy<br>4) tubomanometry<br>5) PTA	No differences in ETDQ-7, TMM, Valsalva and Toynbee maneuvers, tubomanometry, ET score between active and sham surgery arms.	Blinded RCTs are dearly needed to objectively measure the efficacy of BDET.
Hong Ju Park [17]	RCT, prospective, multicenter	n= 121 ears:<br>• BDET+MM (n=62)<br>• MM only (n=59)<br>Follow-up:<br>8 w.	1) ETDQ – 7<br>2) TMM, VM<br>3) PTA	1) ETDQ-7 improvement, BDET vs.MM 6.2 vs.2.6 (p=0.028)<br>2) VM improvement, BDET vs.MM only 46.8% vs. 15.3% (p<0.001)<br>3) ABG decresed<br>BDET > MM only<br>(-5.8±11.4dB) vs.(-1.2±10.5dB)(p=0.023)	BDET combined with MM demonstrated superiority over MM alone.
Ahmed Mohammed Abdelghany [11]	prospective RCT	n=72 ears (subtotal TM perforations+ ETD)<br>• Gr. A: MP+BDET (n=38)<br>• Gr. B: MP only (n=38)<br>Follow-up:<br>24w., 52w.	1) graft take rate<br>2) PTA, ABG<br>3) TMM	1) Graft take rate at 52w. Gr. A vs. B 89.2% vs. 80%<br>2) TMM (mean pressure) Gr. A vs. B<br>at. 24w.: 29 daPa vs. 60 daPa<br>at. 52w.: 55 daPa vs. 79 daPa	BDET is a safe, easy and better in reconstructions of subtotal TM perforations with resistant ETD.

Cheng-Yu Hsieh[16]	RCT	n=50 patients (CRS+OETD):<br>• ESS+BDET (n=25)<br>• ESS (n=25)<br>Follow-up:<br>12 w.	1) SNOT-22<br>2) ETDQ-7<br>3) serial ETF test	1) ETDQ-7 score improvement<br>ESS+BDET vs. ESS<br>12.60 (± 6.50) vs. 6.60 (±5.58) ($p<0.05$)<br>2) ETDQ-7 ratio improvement<br>ESS+BDET vs. ESS<br>92% vs. 68% ($p = 0.034$)<br>3) SNOT-22 score improvement<br>ESS+BDET vs. ESS ($p = 0.78$).	Combined BDET/ESS could decrease otologic symptoms, improve ETF and is appropriate for treating CRS with OETD.
Dong Li[20]	RCT	n=50 patients (subtotal TM perforation):<br>• CNM+BDET(n=25)<br>• CNM only (n=25)<br><br>Follow-up:<br>12 w., 52 w.	1) ETS,<br>2) ETDQ-7,<br>3) ET inflammation scale,<br>4) PTA<br>5) the graft success rate	1) ETDQ-7 score improvement:<br>CNM + BET vs. CNM only<br>at. 12 w.:6.23 vs. 4.22 ($p < 0.01$)<br>at. 52 w.:no significant difference<br>2) the graft success rate<br>CNM + BET vs. CNM only<br>no significant difference at.12 w., 52 w.<br>3) ABG improvement<br>CNM + BET vs. CNM only<br>at. 12 w.:13.16 dB vs.9.74 dB ($p < 0.01$)<br>at.52 w.:no significant difference	BDET combined with CNM had better short-term improvement of hearing and ETDQ-7 scores compared with CNM only.<br>The long-term outcomes were not satisfactory.
Zhou Xu[26]	RCT	n=76 patients AOM<br>• Study group (SG) (BDET + steroid TM inj.) (n=38)<br>• CG (n=38) – steroid TM inj.only	1) proportion of T lymphocytes<br>2) serum inflammatory molecules<br>3) ETS	1) efficacy SG > CG<br>92.1% vs. 68.4% ($p < 0.05$)<br>2) serum inflammatory cytokine levels<br>SG < CG ($p < 0.05$).<br>3) post-treatment ETS, SG vs. CG<br>5.25 vs. 3.53 ($p < 0.05$)	A combination of triamcinolone acetonide and BDET improves efficacy in secretory AOM treatment.

Table S1. Basic characteristics of the studies included in the systematic review

ABG - air-bone gap, PTA - pure-tone audiometry, ET - Eustachian tube, CG - control group, BDET - balloon dilation of the Eustachian Tube, ETD - Eustachian tube dysfunction, ETF - Eustachian tube function, ETS - Eustachian Tube Score, LA - local anesthesia, GA - general anesthesia, TMM – tympanometry, ETDQ-7- Eustachian tube dysfunction questionnaire -7 , VM - Valsalva maneuver, VAS - visual analog scale, TBI - tympanotomy tube insertion, TM - tympanic membranes, RCT- randomized controlled trial, MM - medical management, COME - chronic otitis media with effusion, MP – myringoplasty, ETS - Eustachian tube score, ESS - endoscopic sinus surgery, SNOT-22- sinonasal outcome test 22, CNM - cartilage underlay myringoplasty, AOM – acute secretoty otitis media, inj. - injection

1. Kjaer Krogshede, S., et al., *Balloon Dilation of the Eustachian Tube: A Randomized Controlled Trial with 6 Months Follow-Up.* J Int Adv Otol, 2022. **18**(6): p. 501-506.
2. Dai, B., et al., *Balloon Dilation Eustachian Tuboplasty Combined with Tympanotomy Tube Insertion for Treatment of Refractory Otitis Media with Effusion: A Randomized, Prospective, Controlled Trial Comparing Local Anesthesia Combined Sedation Versus General Anesthesia.* Med Sci Monit, 2023. **29**: p. e938879.
3. Choi, S.W., et al., *A multicenter, randomized, active-controlled, clinical trial study to evaluate the efficacy and safety of navigation guided balloon Eustachian tuboplasty.* Sci Rep, 2021. **11**(1): p. 23296.
4. Poe, D., et al., *Balloon dilation of the eustachian tube for dilatory dysfunction: A randomized controlled trial.* Laryngoscope, 2018. **128**(5): p. 1200-1206.
5. Anand, V., et al., *Balloon Dilation of the Eustachian Tube: 12-Month Follow-up of the Randomized Controlled Trial Treatment Group.* Otolaryngol Head Neck Surg, 2019. **160**(4): p. 687-694.
6. Meyer, T.A., et al., *A Randomized Controlled Trial of Balloon Dilation as a Treatment for Persistent Eustachian Tube Dysfunction With 1-Year Follow-Up.* Otol Neurotol, 2018. **39**(7): p. 894-902.
7. Liang, M., et al., *Effect of the combination of balloon Eustachian tuboplasty and tympanic paracentesis on intractable chronic otitis media with effusion.* Am J Otolaryngol, 2016. **37**(5): p. 442-6.
8. Formankova, D., et al., *Balloon Eustachian Tuboplasty Combined With Tympanocentesis Is not Superior to Balloon Eustachian Tuboplasty in Chronic Otitis Media With Effusion-A Randomized Clinical Trial.* Otol Neurotol, 2020. **41**(3): p. 339-344.
9. Laakso, J.T., et al., *Balloon Eustachian Tuboplasty-A Feasible Double-Blinded Sham Surgery Randomized Clinical Trial Protocol to Study Efficacy.* Laryngoscope, 2024. **134**(4): p. 1874-1881.
10. Kct. A *Prospective Multicenter Randomized Controlled Study for the Evaluation of Efficacy and Safety of Balloon Eustachian Tuboplasty for Patients with Chronic Otitis Media due to Obstructive Tube Dysfunction. https://trialsearch.who.int/Trial2.aspx?TrialID=KCT0005817* **2021**.
11. Abdelghany, A.M. *Does balloon Eustachian tuboplasty increase the success rate in repair of subtotal tympanic membrane perforations with resistant tubal dysfunction? Egyptian Journal of Ear, Nose, Throat and Allied Sciences* **2013**, *14,* 97-101, doi:10.1016/j.ejenta.2013.04.002.
12. Hsieh, C.Y., et al., *Combined balloon Eustachian tuboplasty/endoscopic sinus surgery for patients with chronic rhinosinusitis and Eustachian tube dysfunction.* Int Forum Allergy Rhinol, 2024. **14**(8): p. 1327-1336.
13. Li, D., et al., *Endoscopic cartilage underlay myringoplasty with or without balloon Eustachian tuboplasty for chronic perforation with Eustachian tube dysfunction.* Am J Otolaryngol, 2024. **45**(6): p. 104475.
14. Xu Z, Yan X. Balloon dilation of Eustachian tube combined with triamcinolone acetonide therapy for secretory otitis media. *Tropical Journal of Pharmaceutical Research.* 2024;23(11):1917-1923.
